# Dialysis and Abdominal Defects: Risk of a Massive Hernia When Starting Hemodialysis

**DOI:** 10.7759/cureus.79633

**Published:** 2025-02-25

**Authors:** Daniel Hahn, Lauren Velasquez, Marc R Mohammed, Manu Balasu, Edwin Teehan

**Affiliations:** 1 Internal Medicine, Touro College of Osteopathic Medicine, New York, USA; 2 Internal Medicine, St. Mary's General Hospital, Passaic, USA; 3 General and Vascular Surgery, St. Mary's General Hospital, Passaic, USA

**Keywords:** end-stage renal disease, giant inguinal hernia, hemodialysis complications, hernia repair surgery, mesh inguinal hernioplasty

## Abstract

A giant inguinal hernia is an uncommon clinical challenge, particularly in developed countries where hernias rarely exceed 10 cm in size. In this present case, a 51-year-old male patient with end-stage renal disease (ESRD) on dialysis experienced a 190-pound weight fluctuation, which exacerbated a recurrent inguinal hernia into a giant hernia of 25-30 cm. The patient’s comorbidities of blindness and limited mobility likely contributed to delayed intervention and hernia growth. His surgical management required extensive hernia reduction and a repair with multiple meshes. This case highlights the impact of rapid weight changes on hernia progression and underscores the need for vigilant, interdisciplinary monitoring in high-risk patients.

## Introduction

Inguinal hernias are one of the most common medical conditions in the United States, with approximately 27% of men and 3% of women developing groin, inguinal, or femoral hernias during their lifetime [1]. Inguinal hernias occur when intra-abdominal contents protrude through a weakened inguinal canal or the abdominal wall [2]. Failure of neonatal obliteration of the processus vaginalis, or in adults, the weakening of elastic and collagen fibers leads to the development of inguinal hernias [3].

The two primary classifications of inguinal hernias are either direct or indirect hernias. Direct hernias occur when abdominal contents protrude through weak areas in the posterior wall of the inguinal canal, also known as the Hesselbach triangle. This anatomic triangle is defined laterally by the inferior epigastric vessels, medially by the rectus abdominal muscles, and inferiorly by the inguinal ligament [4]. Indirect hernias are twice as common as direct hernias and occur when abdominal contents enter the deep inguinal ring, pass through the inguinal canal, and exit through the superficial inguinal ring lateral to the inferior epigastric vessels [5]. Indirect hernias lie lateral to the inferior epigastric vessels, while direct hernias lie medial to the inferior epigastric vessels [6]. 

Certain diseases that increase abdominal pressure, such as chronic cough or constipation, or weaken connective tissue, such as Marfan’s or Ehlers-Danlos Syndrome, contribute to an increased incidence of inguinal hernias [5]. Moreover, gender also plays a role in hernia incidence and prevalence. Although global prevalence of hernia saw a 36% absolute rise between 1990 and 2019, males showed a consistent increase in cases while females displayed a more subtle increase [7].

Inguinal hernias are generally treated with surgical repair when there is a risk of strangulation or incarceration of the hernia [8]. When electing to repair inguinal hernias surgically, it is important to have a firm foundation of the surrounding anatomy. Using external landmarks for open repairs, the anterior superior iliac spine of the ilium, the pubic tubercle, and the inguinal ligament are essential for establishing a surgical incision site [4]. Once an incision is made, the abdominal wall layers must be identified until the inguinal canal is reached. These layers, from superficial to deep, are as follows: skin, subcutaneous tissue, Camper’s and Scarpa’s fascias, external oblique fascia and muscle, internal oblique fascia and muscle, transversus abdominis muscle, transversalis fascia, preperitoneal fat, and peritoneum [4]. Once these layers are identified and established, the surgeon will then determine the contents of the myopectineal orifice, the inferior epigastric vessels that form the lateral border of the Hesselbach triangle, and pertinent inguinal nerves that arise from the lumbar plexus to innervate the abdominal muscles and supply sensation to the skin and peritoneum [4]. Appropriate identification of the anatomy surrounding inguinal hernias is a prerequisite for safely proceeding with surgical repair, allowing for fewer complications.

Hernioplasty can be conducted via an open anterior approach or with minimally invasive surgery. While minimally invasive surgeries have become more common recently, most hernias worldwide are still repaired with an open anterior approach [9]. The surgery involves reducing the hernia and then placing a mesh, or if a mesh is contraindicated, a primary suture repair to close the hernia sac [5]. During the hernioplasty procedure, surgeons may opt to perform an antibiotic or antiseptic irrigation of the wound to reduce post-operative complications, such as surgical site infections [10]. Inguinal hernias may also present minimally or asymptomatically; therefore, these can be managed conservatively through “watchful waiting” [5].

The term “giant inguinal hernia" describes a subset of hernias extending below the thigh's midpoint [11]. These hernias are uncommon due to the general practice of early elective repair [11]. One prospective study of 1647 patients found that only 1.1% had a scrotal size greater than 10 cm [8]. Rare cases in literature have found giant hernias between 16 and 30 cm in length. Giant inguinal hernias can present more severe mobility challenges, skin complications such as eczema or ulcers, or potentially recurrent urinary tract infections if there is involvement with the bladder or ureter [11]. In this case, a 51-year-old male patient presented with a massive 30 cm inguinal hernia requiring immediate reduction and repair. Before presenting, the patient had gained 100 pounds and then lost 110 pounds in part due to beginning dialysis less than a year ago, resulting in an enlarging hernia.

## Case presentation

**Figure 1:**
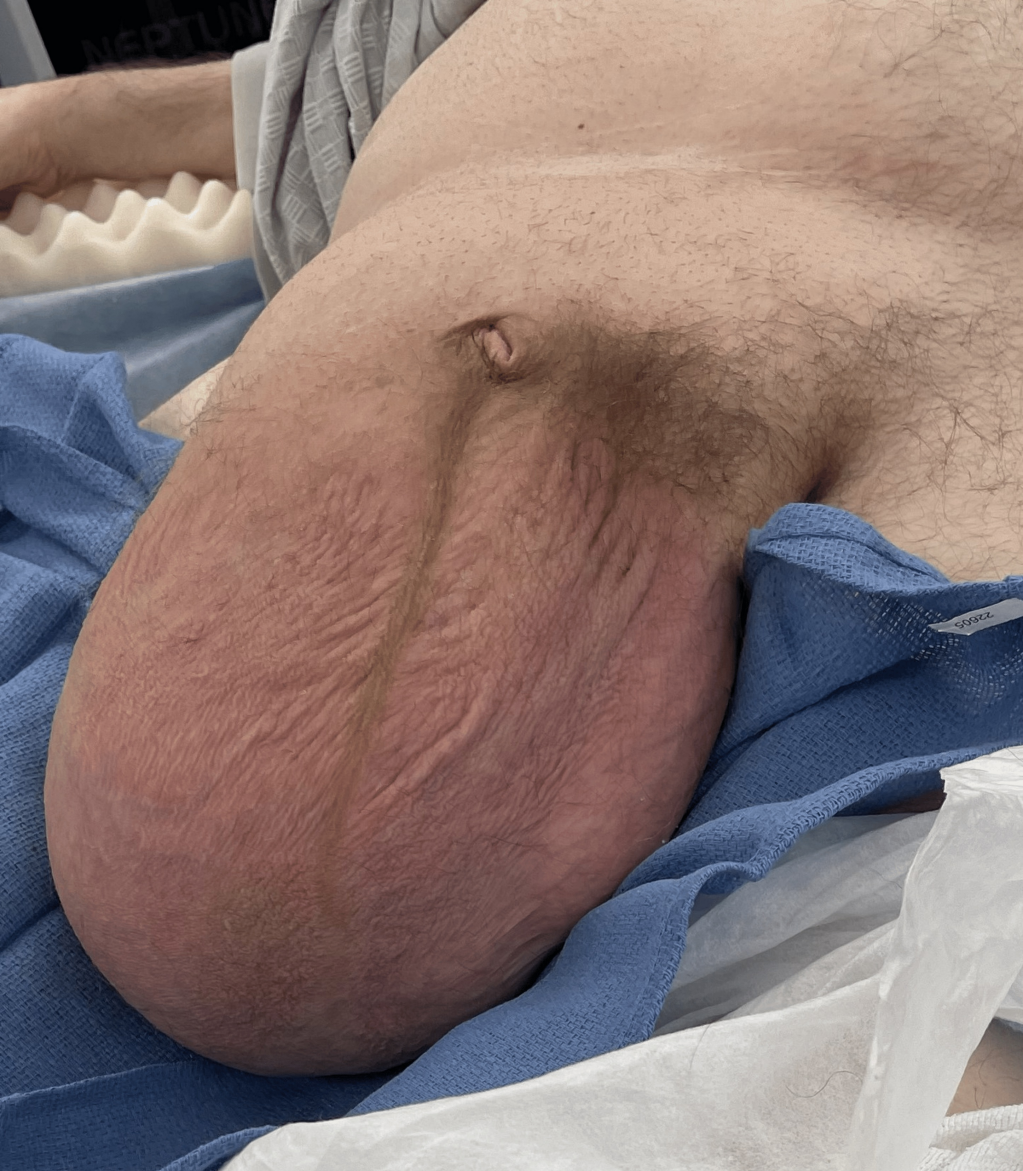
Initial presentation of the giant hernia

A 51-year-old male patient with a past medical history of type 2 diabetes mellitus, hypertension, end-stage renal disease on dialysis, obesity, pulmonary thromboembolism, deep vein thrombosis, and blindness presented with an extremely large, right-sided inguinal hernia in the scrotum (Figure 1). At the time of presentation, the patient was 5’ 8” (172 cm) in height, weighed 255 pounds (116 kg), and had a body mass index (BMI) of 38.8 kg/m^2^. The patient’s scrotum with the hernia extended past the midpoint of his thighs. The patient had a 30-year history of carrying heavy supplies at his retail job. Nine years before the current presentation, the patient had an episode of both an umbilical hernia and a right-sided indirect inguinal hernia, which was operatively fixed with mesh insertions.

The patient first noticed the initial recurrence of the hernia in his right scrotum four years ago. At the time, the patient weighed 260 pounds (118 kg). Since then, the patient gained 100 pounds and weighed 360 pounds (164 kg). Recently, with the initiation of hemodialysis nine months ago, the patient lost 105 pounds, weighing 255 pounds (116 kg). As the hernia got more extensive, the patient stopped walking, frequently fell, and eventually transitioned into a wheelchair. He experienced increased central abdominal pain, constipation, and his penis retracted into the body. The patient reports that the skin over the hernia felt tougher; however, there were no changes in the color, temperature, or feelings of pressure of the hernia. He denies that coughing made the hernia worse.

He does not use tobacco products, drink alcohol, or use illicit drugs. The patient did not have a history of sexually transmitted diseases. He denied nausea, vomiting, diarrhea, changes in his appetite, blood in the stool, changes in the frequency of stools, blood in the urine, changes in the frequency of urination, and no pain with defecation or urination. Due to the acuity of illness and presentation of an incarcerated hernia, the surgery was conducted urgently.

Surgery for hernia

The patient was brought to the operating room with an extremely large scrotum that was 30 cm in length. An incision was made with the scalpel in the right inguinal area, which was much larger than the typical incision. The incision proceeded until the hernia sac was encountered. The hernia sac was located lateral to inferior epigastric arteries, classifying as an indirect hernia, then freed by manually going deep into the scrotum. Due to the size of the contents, the sac could not be reduced. During this process, the incision was enlarged several times. Despite undergoing a prior right-sided indirect inguinal hernioplasty, the previous mesh was either not visualized or not mentioned during the present operation.

An opening was made in the sac, and a long segment of the ileum and cecum was visualized. The bowel was very edematous and swollen. The spermatic cord was separated from the intestinal structures. While trying to reduce the ileum, the size of the opening made the reduction challenging. Additional medial and lateral cauterization was taken to expand the opening further. All of the ileum, then the cecum, the appendix, and the remnant of the omentum were reduced.

The pubic tubercle and the fascia of the Poupart’s ligament inferiorly were examined. Initially, a single 4x6 cm onlay mesh was inserted with interrupted sutures from the pubic tubercle and inferiorly along the Poupart’s ligament. Due to the size of the hernia defect (approximately 3.9x5.5cm), a second 4x6 cm onlay mesh was inserted via healthy-appearing muscle tissue and the Poutpart’s ligament inferiorly. The two meshes were overlapped, and a keyhole was left for the spermatic cord.

**Figure 2:**
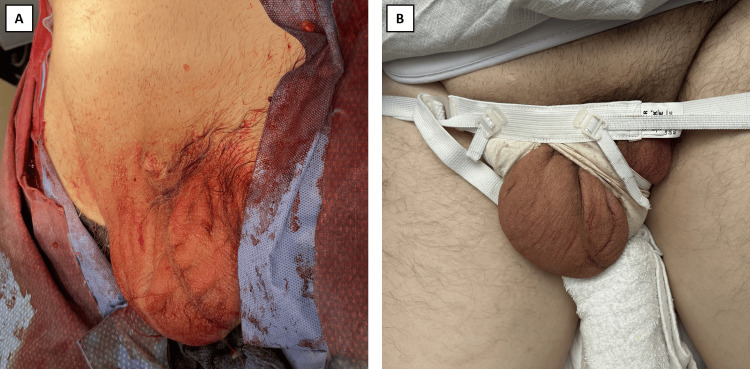
Post-operative recovery of the giant hernia A: Hernia presentation immediately after surgical repair; B: Hernia presentation nine hours after surgical repair

The abdomen and scrotum were irrigated with a diluted Gentamicin and normal saline mixture, and Bupivacaine was injected into the muscle and fascia. The testicle was confirmed inside the scrotum, and the incision was closed (Figure 2A). Scrotal support and an abdominal binder were placed, and the patient left the operating room with normal vital signs. Post-operative mild-to-moderate pain was controlled with acetaminophen and oxycodone/paracetamol. During recovery, the patient's scrotum continued to decrease in size hours after the surgery, and the patient recovered successfully (Figure 2B).

The patient resumed hemodialysis on schedule the day after the surgery. A post-operative physical examination revealed that the patient’s abdomen was soft and non-tender, with a dry dressing intact. The patient denied pain, tolerated oral feeding, was afebrile, and had no nausea or vomiting. Four days after the procedure, the patient was discharged back to his residence.

## Discussion

Inguinal hernias are a common condition where there is displacement of peritoneal content into an adjacent structure, such as the inguinal canal. Giant inguinal hernias are defined by extra-large hernias that descend further than the midpoint of the inner thigh [12]. In limited literature, rare giant hernias tend to be greater than 10 cm in size, and even fewer are greater than 25 cm. Giant inguinal hernias are rare in developed countries; however, when they occur, they are often associated with patients who refuse surgery, may not recognize their emergent size or symptoms, or may have experienced loss of domain from recurrent hernias and hernioplasty procedures. In our patient, because of his history of blindness, recurrent hernias, immobility, and recent fluctuations in weight, he did not recognize the extent of his hernia’s expansion. Physicians should be extra vigilant with patients with a history of recurrent hernias and who may be unable to be watchful of the development of hernias while medically waiting.

Complications of giant hernias can manifest both physically and psychosocially. Hernias have been associated with complex complications, such as abdominal compartment syndrome, torsion of the greater omentum, dehiscence, and acute renal failure due to obstructive uropathy [6,11,12]. Giant inguinal hernias can negatively impact a patient’s daily living activities, mainly when they cause discomfort with walking, sitting, and lying down, as seen with our patient [12].

Hernias are typically caused by conditions that increase intra-abdominal pressure, such as chronic cough or heavy-lifting occupations, or they can be caused by conditions that compromise the extracellular matrix [2,13]. Obesity was previously thought to be a causal factor of hernia development; however, the literature suggests that obese and overweight patients were less likely to develop an inguinal hernia, and obesity may serve as a protective factor. The protective mechanism is believed to result from increased abdominal fat padding, counteracting the weakness of some abdominal areas [2]. Melwani et al. performed a cross-sectional study of 82 patients with an inguinal hernia and found that 86.3% (68/82) patients had normal BMI, and 20.55% (14/82) had low BMI [14]. Another cross-sectional study of 350 patients showed that inguinal hernia repairs were performed primarily on patients with a BMI of 18.5-22 kg/m^2^ (64.2%) compared to those with a BMI of ≥30 kg/m^2^ (1.82%) [13]. Lastly, Rosemar et al. found that in men between the ages 47 and 55 - the same age bracket as our patient - with every increase of 3-4 kg (one BMI unit), the risk of hernias decreased by 4%. Obese men were found to have a 43% lower risk for hernias [15].

There have been nuanced disagreements in the literature about the impact of obesity or weight change being associated with recurrent hernias [2]. It is known that patients with higher BMI tend to have worse post-operative ventral hernia repair outcomes, including wound infection and recurrence [16]. Before hernia repairs, surgeons often encourage weight loss before surgery to improve post-operative outcomes, as lower BMI is associated with lower rates of wound infection, decreased length of stay, and cost of hospitalization. However, Schlosser et al. found that weight loss before hernia repair worsened the hernia, including increased volume and defect parameters. Consequently, increased defects can increase the difficulty of hernia repair [16]. Mechanisms linking worsening hernias to significant weight changes are limited in the literature and require larger scale studies to solidify a correlation between the two.

Several studies suggest that a laparoscopic approach to an inguinal hernia repair in obesity class I and above is comparable to an open approach [17]. This is likely due to the little subcutaneous dissection that occurs during an open inguinal hernia repair. Froylich et al. recommend inguinal hernia repairs should be approached in a method that the surgeon feels comfortable with and is favorable to an obese patient with already increased risk factors for wound morbidity, while still maintaining a durable repair that prevents hernia recurrence [17]. Recurrence rates can be decreased by utilizing a mesh repair, compared to suture repair [18]. Lightweight meshes may have advantages in the short-term post-operative weeks, but long-term data suggests that there are no significant advantages of choosing one mesh type over another in regards to chronic pain [18]. The present case took an open approach with as much conservative dissection allowed to safely reduce the hernia and utilized two onlay meshes.

After starting hemodialysis, end-stage renal disease patients rapidly lose weight, approximately losing 6.5% of their body weight within the initial eight weeks [19]. Banshodani et al. studied 2287 hemodialysis patients in a case-control study and found that a decreased BMI may be a risk factor for inguinal hernia development [20]. The author’s proposed pathogenesis was related to hemodialysis patients with decreased BMI having less abdominal muscle and loose connective tissue in the inguinal area, leading to the formation of the hernia [20]. The present case reports a similar presentation in which the patient experienced 110 pounds of weight loss after initiating hemodialysis treatment, during which the patient noted that the hernia worsened.

## Conclusions

Giant inguinal hernias are massive manifestations of long-standing, untreated hernias that must be addressed to prevent further expansion and complications exacerbated by heavy lifting or, in our present case, rapid weight changes due to hemodialysis. While lowering BMI has post-operative benefits, preoperative weight loss may also lead to worsening of hernia, potentially resulting in complex surgery and recovery. Hernias complicated with hemodialysis require interdisciplinary collaboration to improve outcomes in obese patients who are at risk for rapid weight changes and may need emergency surgery for massive inguinal hernias.
